# Maxillofacial metastasis of genitourinary origin. A report of 3 cases and review of the literature

**DOI:** 10.4317/medoral.17378

**Published:** 2011-12-06

**Authors:** Bartolomé Arias-Chamorro, Francisco Galeas-Anaya, José Salinas-Sánchez, Antonio Acosta-Moyano, Marian Contreras-Morillo, Antonio Valiente-Álvarez, Lucas Bermudo-Añino

**Affiliations:** 1 Senior House Officer, Maxillofacial Surgery Service, Carlos Haya Hospital, Malaga, Spain; 2 Staff Surgeon, Maxillofacial Surgery Service, Carlos Haya Hospital, Malaga, Spain; 3 House Officer, Maxillofacial Surgery Service, Carlos Haya Hospital, Malaga, Spain; 4 Head of Maxillofacial Surgery Service, Carlos Haya Hospital, Malaga, Spain; 5 Head of Management Unit, Maxillofacial Surgery Service, Carlos Haya Hospital, Malaga, Spain

## Abstract

Introduction: The maxillofacial region can harbour a wide range of primary tumours, as well as secondary tumours spreading from distant sites. Rare, though nevertheless important among the latter are genitourinary tumours, such as clear cell renal carcinoma and cervical cancer. Diagnosis of the maxillofacial metastasis sometimes precedes that of the original site, though in other cases the metastasis may arise many years after treatment of the primary tumour. Case report: We present three cases of maxillofacial metastasis of genitourinary tumours, two clear cell renal ade-nocarcinoma and squamous cell carcinoma of the uterine cervix. The patients were referred our hospital service for diagnosis and treatment, after having been initially evaluated for buccodental symptoms. Discussion: The appearance of a maxillofacial tumour, initially with the aspect of a primary tumour, may sometimes be the consequence of haematogenous dissemination from another site, such as these surprising cases originating in the genitourinary area. If disseminated metastatic disease is suspected, an extensive oncological screening should be done to evaluate the best therapeutic option in each patient.

** Key words:** Maxillofacial metastasis, metastatic hypernephroma, cervical cancer.

## Introduction

The maxillofacial area may be host to a wide range of primary tumours, as well as secondary metastatic tumours that have disseminated from distant sites. An uncommon, though nevertheless important group of tumours that can metastasize in this area are those of genitourinary origin (1). Of these, clear cell renal cancer (CCRC) and hypernephroma (Grawitz tumour) account for 3% of all adult neoplasms, and are the third leading cause of infraclavicular tumour metastasizing to the head and neck, after primary tumours of the lung and breast. Metastasis to this area has also been reported from tumours of the prostate, testicles, uterine cervix, skin and digestive tract. CCRC is considered one of the most surprising tumours, due to its highly vascularised explosive growth, with few or no regional symptoms, and which can be associated with synchronic metastasis as the initial manifestation of the disease in distant organs, as occurs in one third of all cases, or metachronic metastasis that can appear many years after nephrectomy for a primary tumour. In order of frequency, this can occur in tumours of the lung, bone, liver, suprarenal gland and brain (2,3).

Worldwide, cervical carcinoma is the third most common type of cancer in women, though in developed countries its incidence has been reduced by programs of early detection and periodical vaginal cytological studies. In advanced stages, cancer of the cervix can disseminate and metastasize, mainly in the lung, bones and liver. Cutaneous metastasis are very unusual, especially in the head and neck, but are a very poor prognostic sign (4). 

These two types of urogenital tumour (CCRC and cancer of the cervix), like others elsewhere in the body, can produce metastasis in the maxillofacial region before their detection at the site of the primary tumour, as we describe here.

## Case Reports

**Figure 1:**
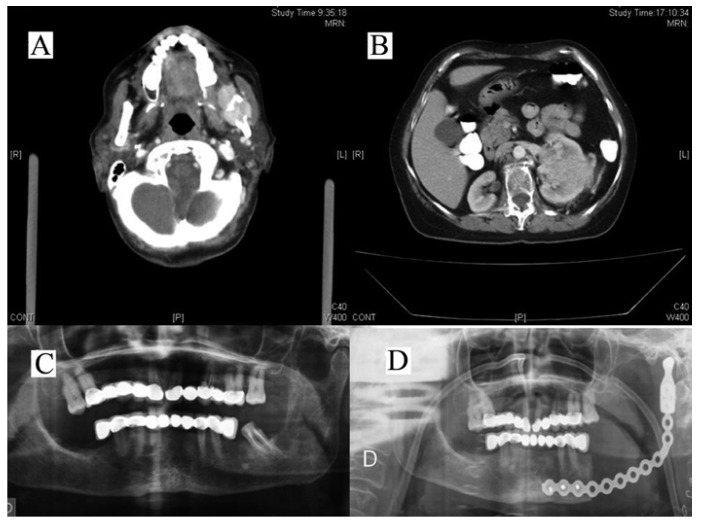
A, B) Cervical and abdominal CT showing synchronic metastasis of a hypernephroma in the left mandibular branch and primary tumour in the left kidney. C, D) Orthopantomography: metastatic radiolucent area in the left mandibular branch and plate reconstruction after hemimandibulectomy.

 -CASE 1: A 62-year-old woman, with no medical history of note, had consulted her dentist on several occasions for a small erythematous sessile lesion behind molar 37, associated with mild pain in the same area. An orthopantomography showed a heterogeneous radiolucent area, apical and distal to the posterior root of molar 37, which had undergone endodontia. Accordingly, the patient was referred to the Maxillofacial Service of our hospital, where she underwent extraction of molar 37 and an incisional biopsy with local anaesthesia of the mucous lesion. Surprisingly, this latter procedure resulted in massive bleeding at the site of the biopsy, and the patient required urgent general anaesthesia for examination and haemostasis of the biopsy site and the left ascending mandibular branch. The biopsy was reported as metastasis of clear cell renal adenocarcinoma. Bone scintigraphy was negative, and oncological orocervical, thoracic and abdominal CT screening were done. The left kidney showed a large 10 cm mass and in the left mandibular angle and branch there was swelling with loss of outline of the osseous cortices, over an irregular radiolucency that reached the sigmoid notch. No other lesions suggestive of metastasis were found. The patient was informed of the nature her disease, and was greatly surprised as she had never experienced any abdominal symptoms. The Oncology Committee recommended left nephrectomy and hemimandibulectomy, which were undertaken at the same time under the same general anaesthesia by the hospital Urology and Maxillofacial Services (Fig. 1). The abdominal post-operative period was uneventful. However, the post-mandibulectomy course was torpid, due to problems of infection on the reconstruction plate and a rib graft. After discharge, the patient was referred to the Medical Oncology Service for chemotherapy. At the time of writing, the patient is free of disease and under regular review by the services involved.

**Figure 2:**
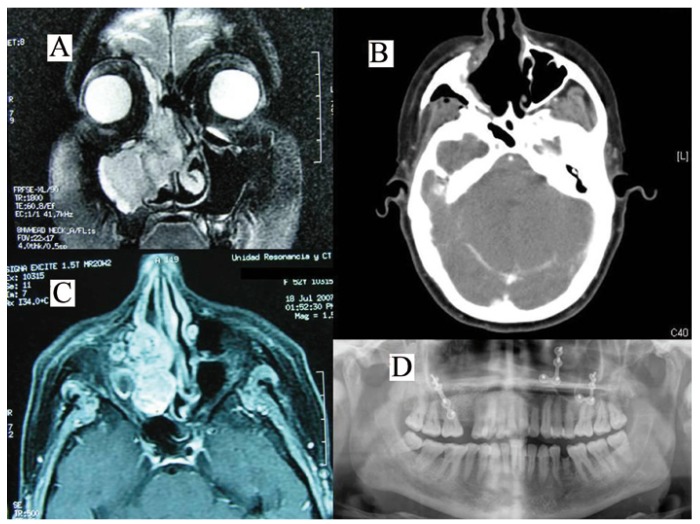
A,C) Craniofacial coronal and axial views showing metachronic metastasis of a previously operated hypernephroma. B, D) Axial CT view and orthopantomography after tumour excision.

 -CASE 2: A 49-year-old woman, with no medical history of note except for a left nephrectomy due to clear cell renal adenocarcinoma 5 years earlier, started experiencing difficulty breathing through the right nare and slight pain in the upper right incisors. The patient was referred to the ENT and Maxillofacial Services for evaluation. Dental and periodontal examination of the relevant zone were normal and the orthopantomography showed a normal maxillary bone and teeth. Nasal fiberoscopy revealed a right nasal polypoid formation in the middle meatus. Biopsy of the lesion resulted in epistaxis requiring an anterior nasal plug to achieve haemostasis. A craniofacial CT study showed occupation of the right maxillary sinus with destruction of the osseous walls, extending to the right nasal fossa, and MRI clearly revealed a 3×2 cm mass in the right nasal fossa. The biopsy was reported as metastasis of CCRC. Oncologic screening with bone scintigraphy and thoracic and abdominal CT was negative. The Oncological Committee recommended resection of the lesion via a Le Fort I surgical approach, after selective angiography of the external carotid artery and embolization of the lesion in order to reduce the risk of operative haemorrhage. A tumorectomy was performed that included the nasal conchae and the lower two thirds of the septum (Fig. 2). The postoperative course was uneventful and the patient received immunotherapy as adjuvant therapy. At the time of writing the patient is disease-free and under regular follow-up.

**Figure 3:**
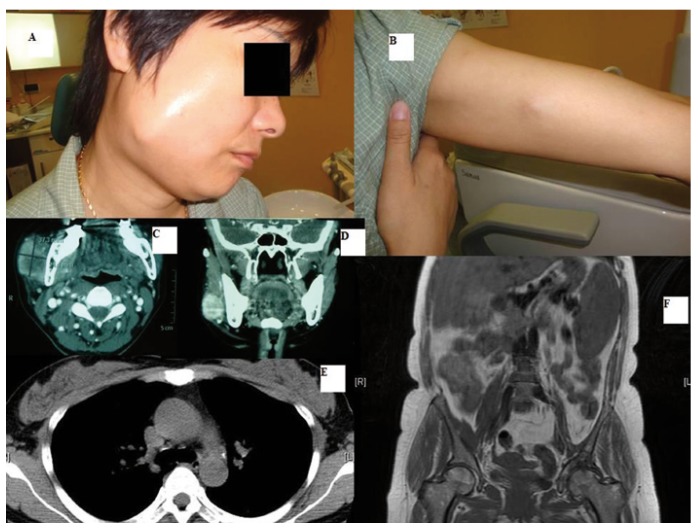
A, B) clinical images of metastases in the right subcutaneous paramandibular region and on the left forearm of a cervical squamous cell carcinoma. C, D) Axial and coronal CT views of the right paramandibular metastasis not involving the skin, mucous or bone. E) Thoracic CT showing small nodules suggestive of pulmonary metastasis. F) Coronal abdominal MR showing squamous cell carcinoma of the cervix, confirmed by biopsy.

 -CASE 3: A 45-year-old Asian woman, with no medical history of note, was referred to the Maxillofacial Service by her dental surgeon, due to the appearance of a progressively enlarging, hard mass in the right mandibular angle, with no initial relation with the neighbouring molars. A biopsy of the lesion was reported as squamous cell carcinoma and a CT showed a mass measuring almost 4 × 2.5 cm in contact with the external mandibular bone cortex. Accordingly, the patient followed the protocol for head and neck oncological surgery. During this period the patient suffered a left renal-ureteral colic requiring hospital admission for parenteral treatment. An abdominal ultrasound study resulted in the casual finding of a 3 cm mass at the confluence of the left ureter and the bladder, with no cleavage plane with the uterine neck. A more exhaustive history and physical examination showed that the patient not only had pain in the area of the facial mass but also pain in the lower abdomen and, more recently, in the left arm in relation with a small recently-appearing nodule. This new information led to replanning of the initial treatment. The biopsy of the arm lesion confirmed the presence of a cutaneous metastasis of squamous cell carcinoma and chest CT and abdominal MRI showed a mass in the uterine neck extending to the bladder and left ureter, with multiple nodules in both right and left hemithorax that, given the history of the patient, were suggestive of metastasis (Fig. 3). Colposcopy showed a mass, biopsy of which resulted in the diagnosis of disseminated squamous cell carcinoma of the uterine cervix. The patient was referred to the Medical Oncology Service for palliative chemotherapy.

## Discussion

Head and neck metastasis of CCRC has been described in the brain, hypophysis, orbit, nasal fossa, ethmoids, oral cavity (gingival), larynx, thyroids and scalp (2,3,5,6). As in our patients, these other cases all appeared synchronically or metachronically with the primary tumour and, depending on the case, with great clinical variation (2,3,7). High vascularisation of the tumour is associated with a high risk of bleeding, either spontaneously or when taking the biopsy. This can cause medical emergencies, which is typical in this type of tumour, as occurred in our cases and has been reported by others (5). Immunohistochemical techniques, such as immunoperoxidase, can be used to distinguish CCRC from other clear cell carcinomas of the salivary glands or of odontogenic origin (5).

Metastasis of cervical cancer in the maxillofacial area has also been reported in the brain, skull, orbit, oral cavity, nasal skin and scalp (4,8-12). In one of our cases we were initially surprised by the presence of a paramandibular squamous cell carcinoma with no causative cutaneous or mucous focus and which was in contact with the mandibular cortex but without involving the bone; this therefore led to doubt about the possibility of an intraosseous carcinoma. The nephritic colic and the biopsied metastasis on the forearm were suggestive of the final diagnosis. 

The cases presented here remind us that the metastatic expression of some tumours may predominate over the primary tumour. Given the wide histological variety of primary and secondary tumours that can metastasize to the maxillofacial area, the origin of a tumour in this area should initially be questioned until a biopsy provides information about the type of tumour and a primary tumour is found to be the obvious cause. Oncological screening is fundamental to provide the most suitable therapeutic option for each patient.

## References

[1] Ogunyemi O, Rojas A, Hematpour K, Rogers D, Head C, Bennett C (2010). Metastasis of genitourinary tumors to the head and neck region. Eur Arch Otorhinolaringol.

[2] Pérez Fentes DA, Blanco Parra M, Toucedo Caamaño J, Lema Grille J, Cimadevila García A, Villar Núñez M (2005). Atypical sites of metastatic renal carcinoma Literature review. Actas Urol Esp.

[3] Makos CP, Psomaderis K (2009). A literature review in renal carcinoma metastasis to the oral mucosa and a new report of an epulis-like metastasis. J Oral Maxillofac Surg.

[4] Chen CH, Chao KC, Wang PH (2007). Advanced cervical squamous cell carcinoma with skin metastasis. Taiwan J Obstet Gynecol.

[5] Will TA, Agarwal N, Petruzzelli GJ (2008). Oral cavity metastasis of renal cell carcinoma: A case report. J Med Case Reports.

[6] Yeh HC, Yang SF, Ke HL, Lee KS, Huang CH, Wu WJ (2007). Renal cell carcinoma presenting with skull metastasis: a case report and literature review. Kaohsiung J Med Sci.

[7] Álvarez-Múgica M, Bulnes Vázquez V, Jalón Monzón A, Gil A, Rodríguez Robles L, Miranda Aranzubía O (2010). Late recurrence from a renal cell carcinoma: solitary right maxilar mass 17 years after surgery. Arch Esp Urol.

[8] Cordeiro JG, Prevedello DM, da Silva Ditzel LF, Pereira CU, Araújo JC (2006). Cerebral metastasis of cervical uterine cancer: report of three cases. Arq Neuropsiquiatr.

[9] Mohanty A, Dutta D, Das S, Samanta D, Senapati S (2010). Skull metastasis from carcinoma of the cervix: A rare case and review of the literature. J Obstet Gynaecol Res.

[10] Lee HM, Choo CT, Poh WT (1997). Orbital metastasis from carcinoma of cervix. Br J Ophthalmol.

[11] Ozdemir H, Tunçbilek G (2009). Metastasis of carcinoma of the uterine cervix to the nasal dorsum. J Craneofac Surg.

[12] Davidson NG, Moyo C (1991). Oral cavity metastasis from carcinoma of the cervix. Int J Gynaecol Obstet.

